# Supplementary material for: GRAF1 integrates PINK1-Parkin signaling and actin dynamics to mediate cardiac mitochondrial homeostasis
Source: Nat Commun. 2023 Dec 11;14:8187. doi: 10.1038/s41467-023-43889-6 (PMC10713658; doi:10.1038/s41467-023-43889-6)
Supplement: Supplementary file 1 — Supplementary Information [file 41467_2023_43889_MOESM1_ESM.pdf]

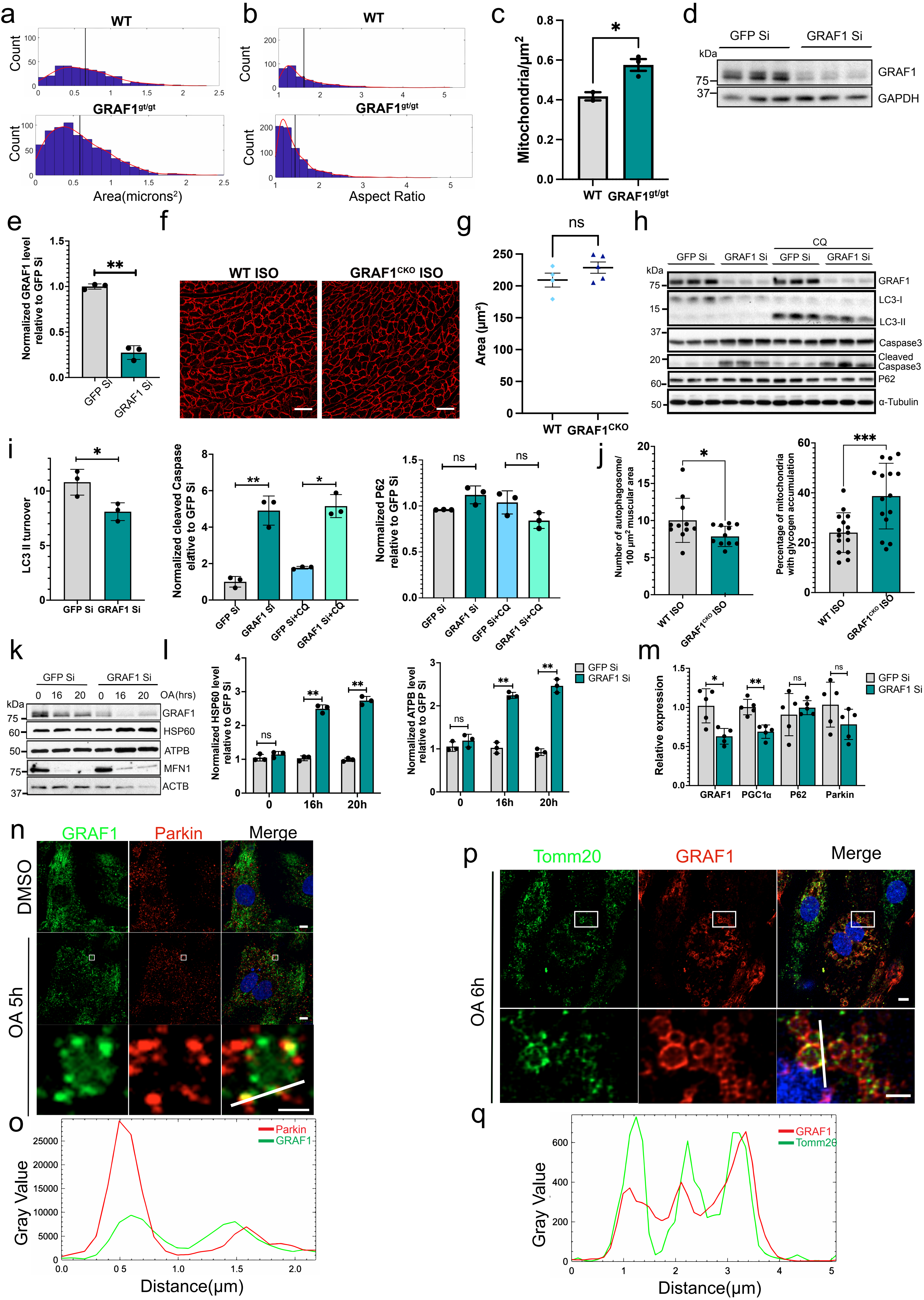

a
WT
Count
GRAF1gt/gt
Area(microns²)
b
WT
Count
GRAF1gt/gt
Aspect Ratio
c
Mitochondria/µm²
*
WT
GRAF1gt/gt
d
GFP Si
GRAF1 Si
kDa
75
37
GRAF1
GAPDH
e
Normalized GRAF1 level relative to GFP Si
**
GFP Si
GRAF1 Si
f
WT ISO
GRAF1CKO ISO
g
ns
Area (µm²)
WT
GRAF1CKO
h
CQ
GFP Si
GRAF1 Si
GFP Si
GRAF1 Si
kDa
GRAF1
LC3-I
LC3-II
Caspase3
Cleaved Caspase3
P62
α-Tubulin
i
LC3 II turnover
GFP Si
GRAF1 Si
Normalized cleaved Caspase relative to GFP Si
GFP Si
GRAF1 Si
GFP Si+CQ
GRAF1 Si+CQ
Normalized P62 relative to GFP Si
ns
j
Number of autophagosome/ 100 µm² muscular area
WT ISO
GRAF1CKO ISO
Percentage of mitochondria with glycogen accumulation
***
k
GFP Si
GRAF1 Si
OA (hrs)
kDa
GRAF1
HSP60
ATPB
MFN1
ACTB
l
Normalized HSP60 level relative to GFP Si
Normalized ATPB level relative to GFP Si
0
16h
20h
GFP Si
GRAF1 Si
m
Relative expression
GRAF1
PGC1α
P62
Parkin
GFP Si
GRAF1 Si
n
GRAF1
Parkin
Merge
DMSO
OA 5h
o
Parkin
GRAF1
Gray Value
Distance(µm)
p
Tomm20
GRAF1
Merge
OA 6h
q
GRAF1
Tomm20
Gray Value
Distance(µm)

Figure S1. GRAF1 promotes cardiomyocyte mitophagy and mitochondrial biogenesis (a-b) Histograms of mitochondrial area and aspect ratio displayed in Fig 1b,c. (a) Histograms (blue bars, 20 bins) overlayed by nonparametric kernel-smoothing distributions (black lines) of mitochondrial cross-sectional areas (CSAs) for TEMs of hearts from wild-type and $GRAF1^{gt/gt}$ mice. (b) Histograms (blue bars, 20 bins) overlayed by nonparametric kernel-smoothing distributions (black lines) of mitochondrial aspect ratios (ARs) for TEMs of hearts from wild-type and $GRAF1^{gt/gt}$ mice. (c) Quantification of mitochondria per μm^2 in TEMs of hearts from wild-type and $GRAF1^{gt/gt}$ mice. Mean mitochondria per μm^2 for wild type = 0.4177 (n = 2), mean mitochondria per μm^2 for $GRAF1^{gt/gt}$ = 0.5755 (n = 3). (d,e) siRNA mediated GRAF1 depletion in NRVCMs was assessed by Western blot (d) and densitometric quantification (e). (f,g) Representative images of laminin-stained heart from WT and $GRAF1^{CKO}$ mice treated with ISO for 14 days.Scale bar:50µm (f). Quantification of cardiomyocyte cross-sectional area (g), n=4-5 mice/group. (h,i) Representative blot (h) and densitometric quantification (i, n=3) showing reduced LC3II turnover, elevated Cleaved Caspase 3, and no change in P62 in GRAF1-deficient cardiomyocytes. 96h post siRNA transfection, NRVCMs were treated with Chloroquine (CQ) 10µM for 4 hours. (j) Quantification of autophagosome number and mitochondria with glycogen accumulation in TEM of hearts from ISO-treated WT and $GRAF1^{CKO}$ mice. See representative TEM Fig 1m. (k,l) Indicated proteins in GRAF1 depleted NRVCM were assessed by Western blot(k) and densitometric analysis was reported, n=3(l). (m) Target genes in siRNA transfected NRVCMs were quantified by real time PCR. n=5. (n) Representative confocal image of endogenous GRAF1 and Parkin in NRVCMs treated with vehicle and OA. Scale bars: 5µm (top) and 1µm (bottom).(o) Intensity profile of GRAF1 and Parkin positive puncta along the white line indicated in (n). (p) Representative confocal image of endogenous GRAF1 and Tomm20 in NRVCMs treated by OA. Scale bars: 5µm (top) and 2µm (bottom). (q) Intensity profile of GRAF1 and Tomm20 along the white line indicated in (p). Data (c,e,g,i,j,l,m) are represented as mean ± SD; ns, not significant;*$P < 0.05$; **$P < 0.01$, ***$P < 0.001$ by two-tailed student's t-test.

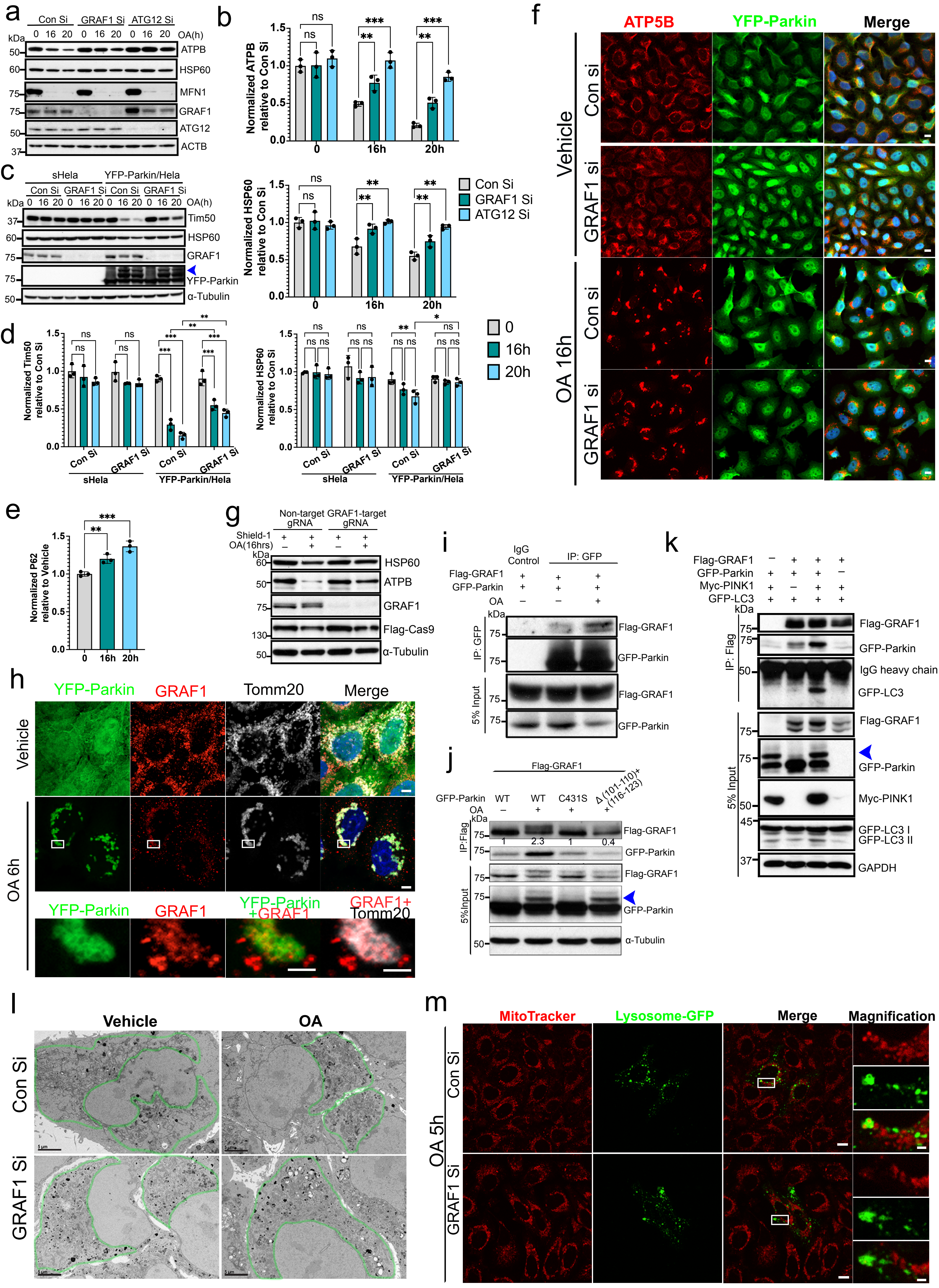
a
Con Si
GRAF1 Si
ATG12 Si
kDa
0 16 20 0 16 20 0 16 20 OA(h)
50
ATPB
60
HSP60
75
MFN1
75
GRAF1
50
ATG12
37
ACTB
b
Normalized ATPB relative to Con Si
ns
***
**
0
16h
20h
c
sHela
YFP-Parkin/Hela
Con Si GRAF1 Si Con Si GRAF1 Si
kDa 0 16 20 0 16 20 0 16 20 0 16 20 OA(h)
37
Tim50
60
HSP60
75
GRAF1
75
YFP-Parkin
50
α-Tubulin
Normalized HSP60 relative to Con Si
Con Si
GRAF1 Si
ATG12 Si
d
Normalized Tim50 relative to Con Si
Normalized HSP60 relative to Con Si
sHela
YFP-Parkin/Hela
0
16h
20h
e
Normalized P62 relative to Vehicle
0
16h
20h
f
ATP5B
YFP-Parkin
Merge
Vehicle
Con si
GRAF1 si
OA 16h
g
Non-target gRNA
GRAF1-target gRNA
Shield-1
OA(16hrs)
kDa
60
HSP60
50
ATPB
75
GRAF1
130
Flag-Cas9
50
α-Tubulin
h
YFP-Parkin
GRAF1
Tomm20
Merge
Vehicle
OA 6h
YFP-Parkin +GRAF1
GRAF1+ Tomm20
i
IgG Control
IP: GFP
Flag-GRAF1
GFP-Parkin
OA
kDa
IP: GFP
5% Input
75
Flag-GRAF1
GFP-Parkin
j
Flag-GRAF1
GFP-Parkin WT WT C431S Δ(101-110)+(116-123)
OA
kDa
IP:Flag
5%Input
1 2.3 1 0.4
Flag-GRAF1
GFP-Parkin
α-Tubulin
k
Flag-GRAF1
GFP-Parkin
Myc-PINK1
GFP-LC3
kDa
IP: Flag
5% Input
Flag-GRAF1
GFP-Parkin
IgG heavy chain
GFP-LC3
Myc-PINK1
GFP-LC3 I
GFP-LC3 II
GAPDH
l
Vehicle
OA
Con Si
GRAF1 Si
5 μm
m
MitoTracker
Lysosome-GFP
Merge
Magnification
OA 5h
Con Si
GRAF1 Si

Figure S2. GRAF1 interacts with Parkin and promotes mitochondrial clustering and association with lysosomes (a,b) siRNA transfected Hela/YFP-Parkin cells were treated with OA for indicated times and mitochondrial proteins were detected and quantified by Western blot/densitometry. (c,d) siRNA transfected sHela and Hela/YFP-Parkin cells were treated by OA for indicated time and mitochondrial protein were assessed by western blot/densitometric quantification. (e) Densitometric quantification of P62 levels following OA treatment. (f)Representative confocal microscopy images showing GRAF1-dependent mitochondrial clearance. Mitochondria were immunostained for ATP5B (see Figure 2c for quantification). Scale bar: 10µm. (g) Representative blot showing defective mitophagy in GRAF1 KO Hela/YFP-Parkin cells. (h) Representative confocal images of YFP-Parkin, endogenous GRAF1 and Tomm20 in Hela/YFP-Parkin cells. Note that a portion of endogenous GRAF1 labeled ring structures colocalize with YFP-Parkin and Tomm20 following OA treatment. Scale bar: 5µm (top), 2µm (magnified images). (i) Reciprocal co-IP of Flag-GRAF1 with GFP-Parkin in COS7 cells transiently expressing GFP-Parkin and Flag-GRAF1 treated by OA for 6 hours. (j) Co-IP of GFP-Parkin WT and its variants with Flag-GRAF1. COS7 cells transiently expressing Flag-GRAF1 and indicated Parkin constructs for 20 hours followed by OA for 6 hours. Δ(101-110)+(116-123) indicates deletion of Parkin amino acids 101-110 and 116-123. (k) Co-IP of GFP-Parkin and GFP-LC3 with Flag-GRAF1. Indicated constructs were co-transfected in COS7 cells for 20 hours. Blue arrowhead (c,j,k) indicates ubiquitinated GFP-Parkin. (l) Representative TEM of Hela/YFP-Parkin cells treated by vehicle or OA for 6 hours. Green lines highlight regions in which mitochondria are localized. (m) Representative confocal image of mitochondria and lysosomes at 5 hours post OA treatment. Hela/Parkin cells were transfected with target siRNA for 48 hours, then were transduced CellLight™ Lysosomes-GFP, BacMam for 20 hours followed by OA treatment for 6 hours. Mitochondria were labeled by Mitotracker Deep Red(200nM) for 30 minutes prior to OA treatment. Scale bar: 10µm. Region of interest (ROI) was magnified and showed on the right. Scale bar: 2µm. Data are represented as mean ± SD; ns, not significant;*$P < 0.05$; **$P < 0.01$, ***$P < 0.001$ by two way ANOVA with Dunnett's post hoc test(b,d) or one way ANOVA with Dunnett's post hoc test(e).

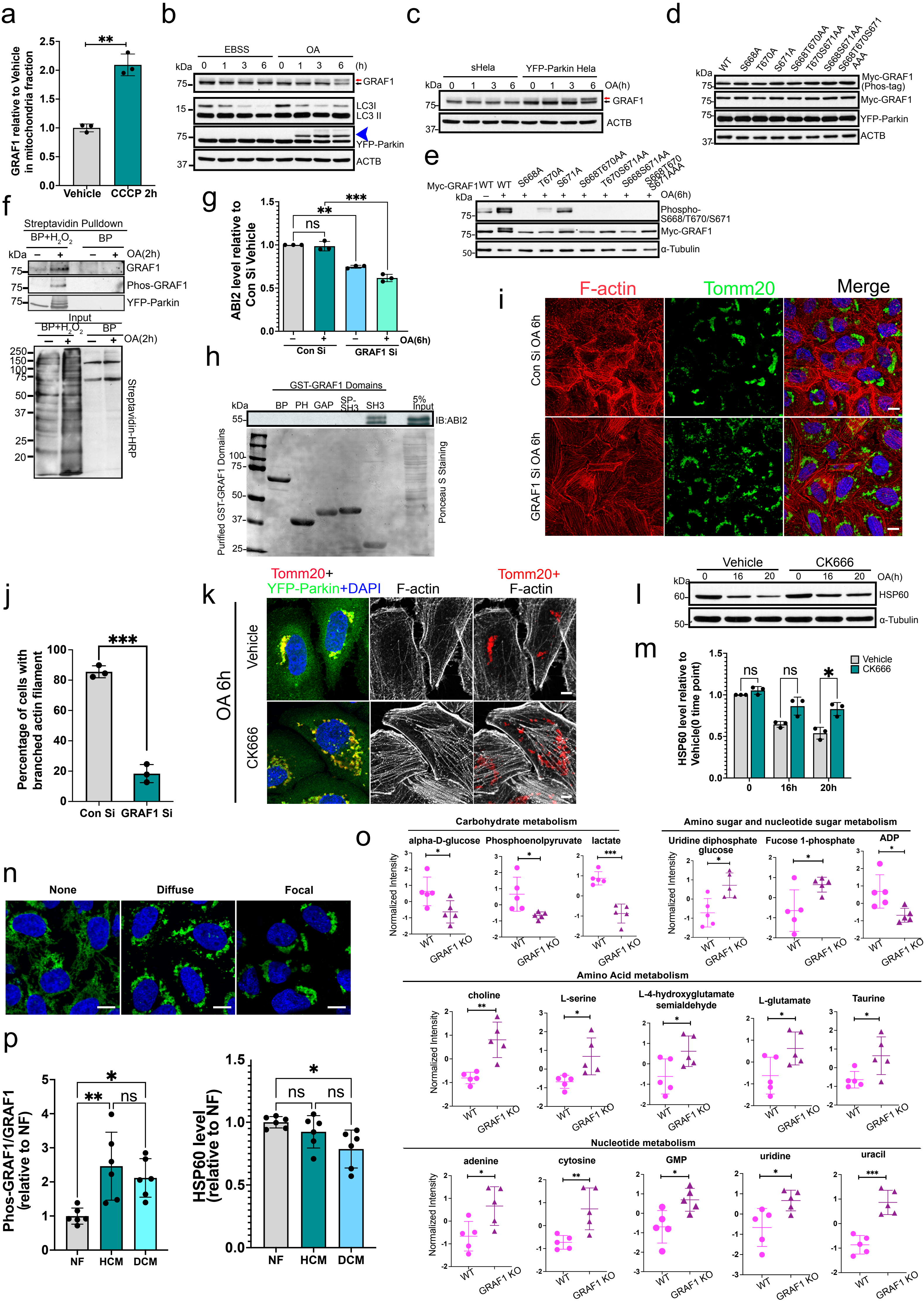

a
GRAF1 relative to Vehicle in mitochondria fraction
Vehicle
CCCP 2h
b
EBSS
OA
kDa
(h)
GRAF1
LC3I
LC3 II
YFP-Parkin
ACTB
c
sHela
YFP-Parkin Hela
OA(h)
GRAF1
ACTB
d
WT
S668A
T670A
S671A
S668T670AA
T670S671AA
S668S671AA
S668T670S671 AAA
Myc-GRAF1 (Phos-tag)
Myc-GRAF1
YFP-Parkin
ACTB
e
Myc-GRAF1
OA(6h)
Phospho-S668/T670/S671
Myc-GRAF1
α-Tubulin
f
Streptavidin Pulldown
BP+H2O2
BP
OA(2h)
GRAF1
Phos-GRAF1
YFP-Parkin
Input
Streptavidin-HRP
g
ABI2 level relative to Con Si Vehicle
ns
OA(6h)
Con Si
GRAF1 Si
h
GST-GRAF1 Domains
BP
PH
GAP
SP-SH3
SH3
5% Input
IB:ABI2
Purified GST-GRAF1 Domains
Ponceau S Staining
i
F-actin
Tomm20
Merge
Con Si OA 6h
GRAF1 Si OA 6h
j
Percentage of cells with branched actin filament
Con Si
GRAF1 Si
k
Tomm20+ YFP-Parkin+DAPI
F-actin
Tomm20+ F-actin
OA 6h
Vehicle
CK666
l
Vehicle
CK666
OA(h)
HSP60
α-Tubulin
m
HSP60 level relative to Vehicle(0 time point)
Vehicle
CK666
ns
16h
20h
n
None
Diffuse
Focal
o
Carbohydrate metabolism
alpha-D-glucose
Phosphoenolpyruvate
lactate
Amino sugar and nucleotide sugar metabolism
Uridine diphosphate glucose
Fucose 1-phosphate
ADP
Normalized Intensity
WT
GRAF1 KO
Amino Acid metabolism
choline
L-serine
L-4-hydroxyglutamate semialdehyde
L-glutamate
Taurine
Nucleotide metabolism
adenine
cytosine
GMP
uridine
uracil
p
Phos-GRAF1/GRAF1 (relative to NF)
HSP60 level (relative to NF)
NF
HCM
DCM

Figure S3 PINK1/Parkin dependent GRAF1 phosphorylation on S668,T670 and S671 facilitates actin remodeling and mitochondrial homeostasis (a) Quantification of mitochondrial-localized GRAF1 on mitochondria in OA-treated Hela/YFP-Parkin cells, see Fig 3a Western blot. (b) Induction of GRAF1 phosphorylation by OA-dependent mitophagy compared to EBSS-induced general autophagy. Blue arrowhead indicates ubiquitinated YFP-Parkin. (c) OA-dependent phosphorylation of GRAF1 in sHela (Parkin deficient) and Hela/YFP-Parkin cells. Red arrow(b,c) denotes phosphorylation-dependent GRAF1 mobility shift. (d) Myc-GRAF1 WT and phosphodeficient variants in Hela/YFP-Parkin were assessed by Western blot. (e) Validation of rabbit anti GRAF1 phos-S668/T670/S671 antibody by Western blot. (f) Hela-Parkin cells expressing APEX2 on the outer membrane of mitochondria were treated with or without OA for 2 hours. Proximity labeling using Biotin-Phenol (BP) and H_20_2 followed by streptavidin immunoprecipitation (IP) and Western blotting for total GRAF1 and pGRAF1. Biotin-labeled proteins in the input were detected using streptavidin-HRP. (g) Densitometric quantification of Co-IP of endogenous ABI2 with endogenous WAVE2, n=3. See Figure 4b Western blot. (h) Purified GST-GRAF1 domains precipitate endogenous ABI2 from Hela/YFP-Parkin cell lysates. Top: ABI2 in pulldown assay and in whole cell lysate. Bottom: purified GST fusion proteins was evaluated by Ponceau S Staining. (i,j) Representative confocal images of Tomm20 and Phalloidin in OA-treated Hela/YFP-Parkin cells. Note continued presence of F-actin bundles and reduced mitochondrial clustering in GRAF1-deficient cells. Quantification of branched actin formation in control and GRAF1-deficinent cells(j). n=3 (≥450 cells/condition). See Figure 4h for representative high magnification images. (k) Representative confocal images of Tomm20, F-actin and YFP-Parkin in Hela/YFP-Parkin cells with or without 100 µM CK666. (l,m) HSP60 in Hela/YFP-Parkin cells following OA treatment with or without 100 µM CK666 and densitometric analysis(m),n=3. (n) Representative images of mitochondrial clustering defined in Fig 4i. (o) Relative normalized intensity of metabolites that significantly differed between ISO-treated WT and $GRAF1^{CKO}$ mouse hearts. (p) Densitometric quantification of phos-GRAF1 and mitochondria mass in human heart samples, see Fig 5o. n=6/group. Scale bar: 10 µm(i,n), 5 µm(k). Data are presented as mean ± SD; ns, not significant; **P* < 0.05; ***P* < 0.01; ****P* < 0.001 by two-tailed student's t-test(a,j,m) or by one way ANOVA with post-hoc Tukey test(g,p).

Table S1. Echocardiographic parameters at different time points

		Basal Level(n=5/group)		7 days post ISO(n=5/group)		14 days post ISO(n=5/group)	
	Unit	WT	GRAF1 KO	WT	GRAF1 KO	WT	GRAF1 KO
**IVS;d**	mm	1.19±0.13	1.15±0.13	1.37±0.1	1.32±0.12	1.31±0.15	1.36±0.1
**IVS;s**	mm	1.94±0.21	1.91±0.08	2.11±0.24	1.97±0.23	2.07±0.16	2.02±0.15
**LVID;d**	mm	3.26±0.5	3.35±0.39	3.45±0.51	3.75±0.43	3.16±0.37	3.68±0.31
**LVID;s**	mm	1.45±0.37	1.48±0.42	1.51±0.68	2.03±0.82	1.13±0.39	1.94±0.27
**LVPW;d**	mm	1.29±0.11	1.32±0.32	1.24±0.14	1.29±0.14	1.47±0.14	1.27±0.15
**LVPW;s**	mm	2±0.1	2.13±0.33	2.19±0.35	2.01±0.31	2.44±0.19	2.06±0.21
**LVEF**	%	87.22±3.99	86.97±6.16	86.63±9.97	76.37±15.9	92.32±4.49	79.16±5.38**
**LVFS**	%	56.21±5.63	56.5±8.34	57.71±13.29	47.26±16.61	64.81±8.81	47.28±5.39*
**Heart rate**	BPM	679.79±30.09	671.48±17.12	692.62±49.99	678.92±26.51	719.55±44.08	712.55±14.49

Mouse Echocardiographic Measurement. Results are presented as mean ± SD, *P =0.023, **P =0.0096, Two-way ANOVA plus Sidak's multiple-comparisons test was used to detect significance. IVS;d, end-diastolic interventricular septal wall thickness. IVS;s, end-systolic interventricular septal wall thickness. LVID;d, left ventricular internal end-diastolic diameter. LVID;s, left ventricular internal end-systolic diameter. LVPW;d, left ventricular end-diastolic posterior wall. LVPW;s, left ventricular end-systolic posterior wall. LVEF, left ventricular ejection fraction. LVFS, left ventricular fractional shortening. BPM: beat per minute.

Table S2. Human patient demographics

ID	Etiology	Gender	Race	Diabetes	HTN	Smoker	CAD	CKD	History of MI	Afib	Prior Cardiac surgery	EF %	LVH	FS %	Meds
50	HCM	M	W	N	N	N	N	N	N	Y	Y; Maze procedure & ICD				
106	HCM	M	B	N	Y	Y	N	Y	Y	N	N	70	Y	14.7	
489	HCM	M	W	N	Y	N	N	Y	N	Y	Y: ICD & VAD	40	Mild	18	
670	HCM	M	W	Y	Y	Y	N	N	N	Y	Y; ICD	45	Severe	36	Carvedilol, milrinone, spironolactone, torsemide
830	HCM	M	W	N	N	N	N	N	N	Y	Y; ICD	35	Mild	25	Dopamine, metolazone, spironolactone, torsemide
1069	HCM	M	Hispanic	N	N	Y	Y	N	Y	Y	Y; ICD, myectomy, CABG	34	Mild	19	Amiodarone, dapagliflozin, metoprolol, entresto, spironolactone
815	DCM	M	W	N	N	N	N	N	N	N	Y; ICD	43	Mild	15	flecainide, sotalol
922	DCM	M	W	Y	Y	Y	Y	N	N	N	Y; CABG, ICD	32	Mild	11	furosemide, isosorbide, mononitrate, metoprolol
973	DCM	M	W	N	N	Y	N	N	N	N	Y; aortic replacement, ICD	14	Moderate	3	furosemide, milrinone
994	DCM	M	B	N	Y	Y	N	Y	N	Y	Y; LVAD, ICD	25	Mild	19	Carvedilol, sildenafil, spironolactone
1037	DCM	M	B	Y	Y	Y	N	N	N	N	Y; LVAD, ICD	20	Mild	10	amiodarone, entresto, bumetanide, carvedilol, empagliflozin
1038	DCM	M	W	N	N	Y	N	N	N	N	N	20	N	6	furosemide, valsartan, spironolactone
59	NF	M	Hispanic	N	N	Y	N	N	N	N	N	55	Mild	31	N
60	NF	M	W	N	N	Y	N	N	N	N	N	35	N	N/A	N
79	NF	M	W	N	Y	Y	N	N	N	N	N	55	Y	29	N
84	NF	M	B	N	N	Y	N	N	N	N	N	50	N	26	N
127	NF	M	W	N	Y	Y	N	N	N	N	N	55	Y	N/A	valsartan
244	NF	M													

HTN: hypertension; CAD: coronary artery disease; CKD: chronic kidney disease; MI: myocardial infarction; Afib: atrial fibrillation; EF%: ejection fraction%; LVH: left ventricular hypertrophy; FS%: fractional shortening%. HCM: hypertrophic cardiomyopathy; DCM: dilated cardiomyopathy; NF: non-failing. HCM patient age: 49.83 ± 4.75; DCM patient age: 45.83 ± 9.7; NF age: 46.67 ± 8.01.
