# Supplementary material for: GRAF1 integrates PINK1-Parkin signaling and actin dynamics to mediate cardiac mitochondrial homeostasis
Source: Nat Commun. 2023 Dec 11;14:8187. doi: 10.1038/s41467-023-43889-6 (PMC10713658; doi:10.1038/s41467-023-43889-6)
Supplement: Supplementary file 3 — Reporting Summary [file 41467_2023_43889_MOESM3_ESM.pdf]

nature portfolio

Corresponding author(s): Joan M. Taylor

Last updated by author(s): 2023/11/20

Reporting Summary

Nature Portfolio wishes to improve the reproducibility of the work that we publish. This form provides structure for consistency and transparency in reporting. For further information on Nature Portfolio policies, see our Editorial Policies and the Editorial Policy Checklist.

Statistics

For all statistical analyses, confirm that the following items are present in the figure legend, table legend, main text, or Methods section.

n/a	Confirmed	
☐	☒	The exact sample size (*n*) for each experimental group/condition, given as a discrete number and unit of measurement
☐	☒	A statement on whether measurements were taken from distinct samples or whether the same sample was measured repeatedly
☐	☒	The statistical test(s) used AND whether they are one- or two-sided<br>*Only common tests should be described solely by name; describe more complex techniques in the Methods section.*
☒	☐	A description of all covariates tested
☐	☒	A description of any assumptions or corrections, such as tests of normality and adjustment for multiple comparisons
☐	☒	A full description of the statistical parameters including central tendency (e.g. means) or other basic estimates (e.g. regression coefficient) AND variation (e.g. standard deviation) or associated estimates of uncertainty (e.g. confidence intervals)
☐	☒	For null hypothesis testing, the test statistic (e.g. *F*, *t*, *r*) with confidence intervals, effect sizes, degrees of freedom and *P* value noted<br>*Give P values as exact values whenever suitable.*
☒	☐	For Bayesian analysis, information on the choice of priors and Markov chain Monte Carlo settings
☒	☐	For hierarchical and complex designs, identification of the appropriate level for tests and full reporting of outcomes
☒	☐	Estimates of effect sizes (e.g. Cohen's *d*, Pearson's *r*), indicating how they were calculated

Our web collection on statistics for biologists contains articles on many of the points above.

Software and code

Policy information about availability of computer code

Data collection	No custom algorithms or software were used herein. TEMs were captured with Gatan Microscopy Suite 3.0 software. For proteomics, LC-MS/MS- the QExactive HF was operated in data-dependent mode where the 15 most intense precursors were selected for subsequent fragmentation. Attune NxT (version 3.1) was used to collect Flow Cytometry data.
Data analysis	GraphPad Prism 10 was used for all statistical analyses. For proteomic analysis, MaxQuant (version 2.4.10.0) was used for grouping and data were imported into Perseus 1.6.14.0 for further processing. Metabolite pathway analysis, hierarchical clustering and heatmap and statistical analysis was conducted using Metaboanalyst 5.0(http://www.metaboanalyst.ca/MetaboAnalyst/). Image Analyses- the freehand selection tool and ROI manager were used to manually define each region of interest in FIJI (version 1.53q). Mitochondrial cross-sectional areas and aspect ratios were plotted in MATLAB (version 9.2.0.959691 (R2017a) as histograms and/or nonparametric kernel-smoothing distributions using hisfit with 20 bins. Colocalization analysis was performed using the Fiji Coloc 2 plugin, which calculated the Mander's colocalization coefficient. FloJo software version 10.6.1 was used to analyze Flow Cytometry data.

For manuscripts utilizing custom algorithms or software that are central to the research but not yet described in published literature, software must be made available to editors and reviewers. We strongly encourage code deposition in a community repository (e.g. GitHub). See the Nature Portfolio guidelines for submitting code & software for further information.

Data

Policy information about availability of data

All manuscripts must include a data availability statement. This statement should provide the following information, where applicable:

- Accession codes, unique identifiers, or web links for publicly available datasets
- A description of any restrictions on data availability
- For clinical datasets or third party data, please ensure that the statement adheres to our policy

All data are available in the main text, supplementary materials or relevant repositories. Proteomics data are available via ProteomeXchange with identifier PXD043212 (https://proteomecentral.proteomexchange.org/) and raw metabolomics data are available on the University of North Carolina Digital Repository with the identifier cr56nb66p (https://cdr.lib.unc.edu/concern/data_sets/cr56nb66p). We will continue our plan to share materials and manage intellectual property, in adherence to the NIH Grant Policy on Sharing of Unique Research Resources including the Sharing of Biomedical Research Resources Principles and Guidelines for Recipients of NIH Grants and Contracts. Plasmids, cell lines and other materials generated in this study are available upon reasonable request to the lead contact. Mouse lines generated by our laboratory will be freely distributed upon assurances detailed below. Some mouse lines produced in the proposed project, however, will be generated by breeding lines previously generated by other laboratories. Thus, following the characterization and peer-reviewed publication of the transgenic mouse strain generated we will either direct requests for mice to the appropriate investigators, or upon receipt of consent from the appropriate investigators we will distribute them to investigators at AAALAC (Association for Assessment and Accreditation of Laboratory Animal Care International) accredited academic institutions wanting mice for non-commercial research. The recipient investigators would provide written assurance and evidence that the animals will be used solely in accord with their local IACAC review; that animals will not be further distributed by the recipient without consent of our program; that animals will not be used for commercial purposes. Requests for mice from for-profit corporations to use the mice commercially will be negotiated by our institution's technology transfer office. All licensing shall be subject to distribution pursuant to my institution's policies and procedures on royalty income. The technology transfer office will report any invention disclosure submitted to them to the appropriate Federal Agency. Should any intellectual property arise which requires a patent, we would ensure that the technology remains widely available to the research community in accordance with the NIH Principles and Guidelines document. Further information and requests for resources and reagents should be directed to and will be fulfilled by the lead contact; Joan Taylor (joan_m_taylor@med.unc.edu).

Research involving human participants, their data, or biological material

Policy information about studies with human participants or human data. See also policy information about sex, gender (identity/presentation), and sexual orientation and race, ethnicity and racism.

Reporting on sex and gender	DHHR approaches everyone undergoing transplantation or LVAD implantation and most everyone consents. Over 70% of such patients admitted to Duke for these purposes are male. Since we were working with 6 samples per group, in order to maximize matching for direct comparison between them we choose to analyze only males for the current study to explore GRAF1 phosphorylation as this parameter was not different in male and female cells.
Reporting on race, ethnicity, or other socially relevant groupings	Participants provided self-reported information regarding race (white, black, hispanic) and data were included for completeness but were not considered as variables for the present study
Population characteristics	Age, sex, and past and current diagnosis (HCM, DCM) were considered important covariates for this study. All males were used with age range of 33-57 and no significant difference in mean age between groups. Additional population characteristics were collected and provided including race, presence of diabetes, HTN, CAD, CKD, Afib, History of MI, and smoking status.
Recruitment	Patients were recruited following admission into the Duke University Hospital System. DHHR approaches everyone undergoing transplantation or LVAD implantation and most everyone consents. LV cardiac tissue from non-failing subjects were obtained from patients who died at Duke Hospital and whose family consented as proxy for them to participate in research through the OPO (organ procurement organization). The OPO them offered the organ to the lab if it was not going to be used for transplantation.
Ethics oversight	Duke University Hospital System Institutional Review Board approved tissue repository (IRB No. Pro00005621)

Note that full information on the approval of the study protocol must also be provided in the manuscript.

Field-specific reporting

Please select the one below that is the best fit for your research. If you are not sure, read the appropriate sections before making your selection.

☒ Life sciences ☐ Behavioural & social sciences ☐ Ecological, evolutionary & environmental sciences

For a reference copy of the document with all sections, see nature.com/documents/nr-reporting-summary-flat.pdf

Life sciences study design

All studies must disclose on these points even when the disclosure is negative.

Sample size	All in vitro data were from three to five ndependent experiments without pre-determination based on prior studies in the lab or in the literature. For animal studies, sample size calculation was performed based on prior studies and power analyses using an expected difference in means of 25% and a power of 0.8
Data exclusions	For proteomics, only proteins with >1 unique+razor peptide were used for LFQ analysis Results were filtered to 1% FDR at the unique peptide level. For metabolomics, To avoid infinite values when calculating fold changes, a value 1000 was used for metabolites not detected in some samples but that had measurable signals in one or more other samples.
Replication	Reproducibility was verified by combining data from at least 3 independent experiments. No experiments were discarded.
Randomization	All experiments contained all samples to be compared in each dataset.
Blinding	Image collection was performed randomly (i.e. 5 fields per slide). All experimentalists scoring images for analysis were blinded to sample name. Animal experiments were also performed in a blinded fashion to the investigator performing the surgery or the echocardiography. Likewise, metabolomics and proteomics data were generated and analyzed by investigators blinded to sample names. Blinding was not performed in other biochemical or cell biology experiments since quantitative outputs were generated by relevant technological equipment.

Behavioural & social sciences study design

All studies must disclose on these points even when the disclosure is negative.

Study description	*Briefly describe the study type including whether data are quantitative, qualitative, or mixed-methods (e.g. qualitative cross-sectional, quantitative experimental, mixed-methods case study).*
Research sample	*State the research sample (e.g. Harvard university undergraduates, villagers in rural India) and provide relevant demographic information (e.g. age, sex) and indicate whether the sample is representative. Provide a rationale for the study sample chosen. For studies involving existing datasets, please describe the dataset and source.*
Sampling strategy	*Describe the sampling procedure (e.g. random, snowball, stratified, convenience). Describe the statistical methods that were used to predetermine sample size OR if no sample-size calculation was performed, describe how sample sizes were chosen and provide a rationale for why these sample sizes are sufficient. For qualitative data, please indicate whether data saturation was considered, and what criteria were used to decide that no further sampling was needed.*
Data collection	*Provide details about the data collection procedure, including the instruments or devices used to record the data (e.g. pen and paper, computer, eye tracker, video or audio equipment) whether anyone was present besides the participant(s) and the researcher, and whether the researcher was blind to experimental condition and/or the study hypothesis during data collection.*
Timing	*Indicate the start and stop dates of data collection. If there is a gap between collection periods, state the dates for each sample cohort.*
Data exclusions	*If no data were excluded from the analyses, state so OR if data were excluded, provide the exact number of exclusions and the rationale behind them, indicating whether exclusion criteria were pre-established.*
Non-participation	*State how many participants dropped out/declined participation and the reason(s) given OR provide response rate OR state that no participants dropped out/declined participation.*
Randomization	*If participants were not allocated into experimental groups, state so OR describe how participants were allocated to groups, and if allocation was not random, describe how covariates were controlled.*

Ecological, evolutionary & environmental sciences study design

All studies must disclose on these points even when the disclosure is negative.

Study description	*Briefly describe the study. For quantitative data include treatment factors and interactions, design structure (e.g. factorial, nested, hierarchical), nature and number of experimental units and replicates.*
Research sample	*Describe the research sample (e.g. a group of tagged Passer domesticus, all Stenocereus thurberi within Organ Pipe Cactus National Monument), and provide a rationale for the sample choice. When relevant, describe the organism taxa, source, sex, age range and any manipulations. State what population the sample is meant to represent when applicable. For studies involving existing datasets, describe the data and its source.*
Sampling strategy	*Note the sampling procedure. Describe the statistical methods that were used to predetermine sample size OR if no sample-size calculation was performed, describe how sample sizes were chosen and provide a rationale for why these sample sizes are sufficient.*

Data collection	*Describe the data collection procedure, including who recorded the data and how.*
Timing and spatial scale	*Indicate the start and stop dates of data collection, noting the frequency and periodicity of sampling and providing a rationale for these choices. If there is a gap between collection periods, state the dates for each sample cohort. Specify the spatial scale from which the data are taken*
Data exclusions	*If no data were excluded from the analyses, state so OR if data were excluded, describe the exclusions and the rationale behind them, indicating whether exclusion criteria were pre-established.*
Reproducibility	*Describe the measures taken to verify the reproducibility of experimental findings. For each experiment, note whether any attempts to repeat the experiment failed OR state that all attempts to repeat the experiment were successful.*
Randomization	*Describe how samples/organisms/participants were allocated into groups. If allocation was not random, describe how covariates were controlled. If this is not relevant to your study, explain why.*
Blinding	*Describe the extent of blinding used during data acquisition and analysis. If blinding was not possible, describe why OR explain why blinding was not relevant to your study.*

Did the study involve field work? ☐ Yes ☐ No

Field work, collection and transport

Field conditions	*Describe the study conditions for field work, providing relevant parameters (e.g. temperature, rainfall).*
Location	*State the location of the sampling or experiment, providing relevant parameters (e.g. latitude and longitude, elevation, water depth).*
Access & import/export	*Describe the efforts you have made to access habitats and to collect and import/export your samples in a responsible manner and in compliance with local, national and international laws, noting any permits that were obtained (give the name of the issuing authority, the date of issue, and any identifying information).*
Disturbance	*Describe any disturbance caused by the study and how it was minimized.*

Reporting for specific materials, systems and methods

We require information from authors about some types of materials, experimental systems and methods used in many studies. Here, indicate whether each material, system or method listed is relevant to your study. If you are not sure if a list item applies to your research, read the appropriate section before selecting a response.

Materials & experimental systems

n/a	Involved in the study	
☐	☒	Antibodies
☐	☒	Eukaryotic cell lines
☒	☐	Palaeontology and archaeology
☐	☒	Animals and other organisms
☒	☐	Clinical data
☒	☐	Dual use research of concern
☒	☐	Plants

Methods

n/a	Involved in the study	
☒	☐	ChIP-seq
☐	☒	Flow cytometry
☒	☐	MRI-based neuroimaging

Antibodies

Antibodies used	The following primary antibodies were used for Western blotting: mouse anti-SQSTM1/p62 (ab56416, Abcam, 1:3000), rabbit anti-LC3A/B (12741S, Cell Signaling Technology, 1:1000), mouse anti-Flag M5(F4042, Sigma, 1:4000), rabbit anti-Myc(2278S, Cell Signaling Technology, 1:1000), mouse anti-Parkin(4211S, Cell Signaling Technology, 1:1000), rabbit anti-PINK1(6946S, Cell Signaling Technology, 1:500), rabbit anti-GFP(A11122, Thermo Fisher, 1:1000), mouse anti-β-Actin(3700S, Cell Signaling Technology, 1:1000), rabbit anti-GAPDH(5174S, Cell Signaling Technology, 1:1000), mouse anti-α-Tubulin(T6074, Sigma, 1:3000), rabbit anti-HSP60 (12165S, Cell Signaling Technology, 1:1000), rabbit anti-VDAC(4661S, Cell Signaling Technology, 1:1000), rabbit anti-ATG7(8558S, Cell Signaling Technology,1:500), mouse anti-TIM50(sc-393678, Santa Cruz,1:1000), rabbit anti-PHB2(PA5-14133, Thermo Fisher, 1:500), rabbit anti-ATPB(ab14730, Abcam,1:500), rabbit anti-WAVE-2(3659S, Cell Signaling Technology, 1:1000), rabbit anti-ABI2(14890-1-AP, Proteintech, 1:500), rabbit anti-CYFIP1(ab156016, Abcam, 1:500). Rabbit anti-GRAF1 and rabbit anti-GRAF1 phospho-S668T670S671 polyclonal antibodies were homemade antibodies in our lab. The following antibodies and concentrations were used for immunocytochemistry : Primary antibodies were mouse monoclonal anti-ATP5B (MAB3494, Millipore, 1:500), rabbit anti-HSP60 (12165S, Cell Signaling Technology, 1:500), mouse anti-TOMM20 (sc-17764, Santa Cruz, 1:250), mouse anti-LC3B(sc-398822, Santa Cruz, 1:100), mouse anti-HSP60(sc-13115, Santa Cruz, 1:250), chicken anti-GFP(A10262, Thermo Fisher, 1:500), rabbit anti-GRAF1 (homemade antibody, 1:250). Secondary antibodies were highly cross-adsorbed goat anti-mouse Alexa Fluor 488(A-11001, Thermo

Fisher, 1:500), goat anti-rabbit Alexa Fluor 488(A11008, Thermo Fisher, 1:500), donkey anti-rabbit Alexa Fluor 555(A31570, Thermo Fisher, 1:500), and donkey anti-mouse Alexa Fluor 647(A31571, Thermo Fisher, 1:500), goat anti-chicken Alexa Fluor 488(A11039, Thermo Fisher, 1:500). F-actin was stained by Phalloidin Alexa Fluor 555(A34055, Thermo Fisher, 1:100). The following antibodies were used for immunohistochemistry; primary antibodies included mouse anti MYH6(Ab207926, Abcam, 1:250), rabbit anti-HSP60 (12165S, Cell Signaling Technology, 1:250), Secondary antibodies were highly cross-adsorbed goat anti-mouse Alexa Fluor 488 (A-11001, Thermo Fisher,1:500), goat anti-rabbit Alexa Fluor 555(A-21428, Thermo Fisher,1:500).

Validation

We only use antibodies from reputable sources (e.g. MilliporeSigma, Cell Signaling Technology, or Abcam) that are accompanied both by validation experiments on company website and lists of references that we refer to prior to ordering antibody. Upon receipt, we rigorously test them by Western blotting and immunofluorescence using positive controls (in vitro translated protein) and negative controls (knockout or knockdown cell lines). Phospho-specific antibodies have been validated by pre-treatment of samples with phosphatases. Antibodies that have previously been validated in the literature are preferred. Depending on availability, monoclonal antibodies are preferred over their polyclonal counterparts to exploit their specificity and minimal to no batch-to-batch variability.

Eukaryotic cell lines

Policy information about cell lines and Sex and Gender in Research

Cell line source(s)

Hela cells –Hela cells expressing mCherry-Parkin, YFP-LC3/mCherry-Parkin Hela cells, YFP-Parkin Hela cells and mCherry-Parkin Hela cells (female) were all obtained from Richard Youle (NIH) and prepared as described in the manuscript entitled "The ubiquitin kinase PINK1 recruits autophagy receptors to induce mitophagy" published in Nature, 2015. Cos7 cells (male) were from ATCC. Primary cardiomyocytes were isolated from neonatal male and female rats.

Authentication

For cell lines, perform short tandem repeat (STR) profiling on these line every 4 months and if cells do not match the original STR profile by at least 80%, we return to one of our original thaws. For cardiomyocytes, each primary preparation is analyzed for cardiac differentiation markers (alpha MHC and troponin T) and for fibroblast markers (FSP-1 and vimentin etc.). Only those preparations that are >95% alpha MHC positive AND FSP-1 negative are used. Since these cells are usually not passaged there is no need for STR profiling.

Mycoplasma contamination

We use an accurate, sensitive, and reliable PCR detection method to test all cultures for mycoplasma contamination every few months. All cells used for these studies tested negative for mycoplasma.

Commonly misidentified lines
(See ICLAC register)

no commonly mis-identified cells have been used in this study

Palaeontology and Archaeology

Specimen provenance

Provide provenance information for specimens and describe permits that were obtained for the work (including the name of the issuing authority, the date of issue, and any identifying information). Permits should encompass collection and, where applicable, export.

Specimen deposition

Indicate where the specimens have been deposited to permit free access by other researchers.

Dating methods

If new dates are provided, describe how they were obtained (e.g. collection, storage, sample pretreatment and measurement), where they were obtained (i.e. lab name), the calibration program and the protocol for quality assurance OR state that no new dates are provided.

☐ Tick this box to confirm that the raw and calibrated dates are available in the paper or in Supplementary Information.

Ethics oversight

Identify the organization(s) that approved or provided guidance on the study protocol, OR state that no ethical approval or guidance was required and explain why not.

Note that full information on the approval of the study protocol must also be provided in the manuscript.

Animals and other research organisms

Policy information about studies involving animals; ARRIVE guidelines recommended for reporting animal research, and Sex and Gender in Research

Laboratory animals

NRVCMs were isolated from 2-3 day old male and female Wistar rats. All mice including CAG-FLPe deleter mice were on a C57Black6/J background. Adult males 10-12 weeks of age were used for experiments, animals were bred between 3 mo and 9 mo of age. Mice were housed at temperatures of 20-23°C with 40-60% humidity.

Wild animals

no wild animals were used in the study

Reporting on sex

only male mice were used, but prior published studies determined lack of sexual dimorphism in cardiac outcomes in GRAF1-deficient animals and ISO treated WT mice. This limitation was discussed.

Field-collected samples

no field-collected samples were used in the study

Ethics oversight

UNC IACUC; UNC IRB; Duke University Hospital Institutional Review Board

Note that full information on the approval of the study protocol must also be provided in the manuscript.

Clinical data

Policy information about clinical studies

All manuscripts should comply with the ICMJEguidelines for publication of clinical research and a completedCONSORT checklist must be included with all submissions.

Clinical trial registration	*Provide the trial registration number from ClinicalTrials.gov or an equivalent agency.*
Study protocol	*Note where the full trial protocol can be accessed OR if not available, explain why.*
Data collection	*Describe the settings and locales of data collection, noting the time periods of recruitment and data collection.*
Outcomes	*Describe how you pre-defined primary and secondary outcome measures and how you assessed these measures.*

Dual use research of concern

Policy information about dual use research of concern

Hazards

Could the accidental, deliberate or reckless misuse of agents or technologies generated in the work, or the application of information presented in the manuscript, pose a threat to:

No	Yes	
☒	☐	Public health
☒	☐	National security
☒	☐	Crops and/or livestock
☒	☐	Ecosystems
☒	☐	Any other significant area

Experiments of concern

Does the work involve any of these experiments of concern:

No	Yes	
☒	☐	Demonstrate how to render a vaccine ineffective
☒	☐	Confer resistance to therapeutically useful antibiotics or antiviral agents
☒	☐	Enhance the virulence of a pathogen or render a nonpathogen virulent
☒	☐	Increase transmissibility of a pathogen
☒	☐	Alter the host range of a pathogen
☒	☐	Enable evasion of diagnostic/detection modalities
☒	☐	Enable the weaponization of a biological agent or toxin
☒	☐	Any other potentially harmful combination of experiments and agents

Plants

Seed stocks	*Report on the source of all seed stocks or other plant material used. If applicable, state the seed stock centre and catalogue number. If plant specimens were collected from the field, describe the collection location, date and sampling procedures.*
Novel plant genotypes	*Describe the methods by which all novel plant genotypes were produced. This includes those generated by transgenic approaches, gene editing, chemical/radiation-based mutagenesis and hybridization. For transgenic lines, describe the transformation method, the number of independent lines analyzed and the generation upon which experiments were performed. For gene-edited lines, describe the editor used, the endogenous sequence targeted for editing, the targeting guide RNA sequence (if applicable) and how the editor was applied.*
Authentication	*Describe any authentication procedures for each seed stock used or novel genotype generated. Describe any experiments used to assess the effect of a mutation and, where applicable, how potential secondary effects (e.g. second site T-DNA insertions, mosiacism, off-target gene editing) were examined.*

ChIP-seq

Data deposition

☐ Confirm that both raw and final processed data have been deposited in a public database such as GEO.

☐ Confirm that you have deposited or provided access to graph files (e.g. BED files) for the called peaks.

Data access links<br>*May remain private before publication.*	*For "Initial submission" or "Revised version" documents, provide reviewer access links. For your "Final submission" document, provide a link to the deposited data.*
Files in database submission	*Provide a list of all files available in the database submission.*
Genome browser session<br>(e.g. UCSC)	*Provide a link to an anonymized genome browser session for "Initial submission" and "Revised version" documents only, to enable peer review. Write "no longer applicable" for "Final submission" documents.*

Methodology

Replicates	*Describe the experimental replicates, specifying number, type and replicate agreement.*
Sequencing depth	*Describe the sequencing depth for each experiment, providing the total number of reads, uniquely mapped reads, length of reads and whether they were paired- or single-end.*
Antibodies	*Describe the antibodies used for the ChIP-seq experiments; as applicable, provide supplier name, catalog number, clone name, and lot number.*
Peak calling parameters	*Specify the command line program and parameters used for read mapping and peak calling, including the ChIP, control and index files used.*
Data quality	*Describe the methods used to ensure data quality in full detail, including how many peaks are at FDR 5% and above 5-fold enrichment.*
Software	*Describe the software used to collect and analyze the ChIP-seq data. For custom code that has been deposited into a community repository, provide accession details.*

Flow Cytometry

Plots

Confirm that:

☒ The axis labels state the marker and fluorochrome used (e.g. CD4-FITC).

☒ The axis scales are clearly visible. Include numbers along axes only for bottom left plot of group (a 'group' is an analysis of identical markers).

☒ All plots are contour plots with outliers or pseudocolor plots.

☒ A numerical value for number of cells or percentage (with statistics) is provided.

Methodology

Sample preparation	96 hours following indicated siRNA transfection, NRVCMs were trypsinized from 100 mm dishes and passed through 70µm mesh size cell strainer (Falcon) to remove cell aggregates. Next, NRVCMs were aliquoted at approximate 3x105 /ml in Eppendorf tube and incubated with JC-1(2µM) in the presence or absence of CCCP (50µM) for 30 minutes at 37°C and 5% CO2. NRVCMS then were quickly washed 2 times with PBS prior to subjecting to flow cytometry.
Instrument	Attune NxT flow cytometer(Thermo Fisher)
Software	Attune NxT software was used to collect data and FlowJo was used to analyze data
Cell population abundance	1x105 NRVCMs of each experimental condition were recorded

Gating strategy

Firstly, unstained cell samples were run to set up appropriate PMT (FSC and SSC) voltages. The goal was to adjust the voltages to ensure most cells, if not all, were within the plot. It was important to observe that the fluorescent signal of unstained cells was not positive, indicating that it did not exceed the background noise on the histogram. In the bivariate plot, the unstained sample should have appeared in the down-left region. Next, a small amount of staining samples was run, using green and red fluorescence channels. The samples were acquired at a rate of 25ul per minute, with 10000 events in a 50ul acquisition volume. This step helped to establish gating strategies and determine optimal PMT voltages for each fluorescence channel. The gating process involved creating dot plots. The first dot plot used FSC-A (x-axis) and SSC-A (y-axis) to gate out cell debris. Appropriate gates were set to exclude events associated with debris. The second dot plot used FSC-A (x-axis) and FSC-H (y-axis) to identify and exclude doublet cells, ensuring the focus remained on singlet cells. Adjustments were made to the gating strategy to exclude events falling outside the singlet region. Finally, a dot plot was created using green-A (x-axis) and red-A (y-axis) to visualize cells stained with JC-1. This plot facilitated the observation of cell populations based on JC-1 staining.

☐ Tick this box to confirm that a figure exemplifying the gating strategy is provided in the Supplementary Information.

Magnetic resonance imaging

Experimental design

Design type

Indicate task or resting state; event-related or block design.

Design specifications

Specify the number of blocks, trials or experimental units per session and/or subject, and specify the length of each trial or block (if trials are blocked) and interval between trials.

Behavioral performance measures

State number and/or type of variables recorded (e.g. correct button press, response time) and what statistics were used to establish that the subjects were performing the task as expected (e.g. mean, range, and/or standard deviation across subjects).

Acquisition

Imaging type(s)

Specify: functional, structural, diffusion, perfusion.

Field strength

Specify in Tesla

Sequence & imaging parameters

Specify the pulse sequence type (gradient echo, spin echo, etc.), imaging type (EPI, spiral, etc.), field of view, matrix size, slice thickness, orientation and TE/TR/flip angle.

Area of acquisition

State whether a whole brain scan was used OR define the area of acquisition, describing how the region was determined.

Diffusion MRI ☐ Used ☐ Not used

Preprocessing

Preprocessing software

Provide detail on software version and revision number and on specific parameters (model/functions, brain extraction, segmentation, smoothing kernel size, etc.).

Normalization

If data were normalized/standardized, describe the approach(es): specify linear or non-linear and define image types used for transformation OR indicate that data were not normalized and explain rationale for lack of normalization.

Normalization template

Describe the template used for normalization/transformation, specifying subject space or group standardized space (e.g. original Talairach, MNI305, ICBM152) OR indicate that the data were not normalized.

Noise and artifact removal

Describe your procedure(s) for artifact and structured noise removal, specifying motion parameters, tissue signals and physiological signals (heart rate, respiration).

Volume censoring

Define your software and/or method and criteria for volume censoring, and state the extent of such censoring.

Statistical modeling & inference

Model type and settings

Specify type (mass univariate, multivariate, RSA, predictive, etc.) and describe essential details of the model at the first and second levels (e.g. fixed, random or mixed effects; drift or auto-correlation).

Effect(s) tested

Define precise effect in terms of the task or stimulus conditions instead of psychological concepts and indicate whether ANOVA or factorial designs were used.

Specify type of analysis: ☐ Whole brain ☐ ROI-based ☐ Both

Statistic type for inference

(See Eklund et al. 2016)

Specify voxel-wise or cluster-wise and report all relevant parameters for cluster-wise methods.

Correction | *Describe the type of correction and how it is obtained for multiple comparisons (e.g. FWE, FDR, permutation or Monte Carlo).*

Models & analysis

n/a	Involved in the study
☐	☐ Functional and/or effective connectivity
☐	☐ Graph analysis
☐	☐ Multivariate modeling or predictive analysis

Functional and/or effective connectivity | *Report the measures of dependence used and the model details (e.g. Pearson correlation, partial correlation, mutual information).*

Graph analysis | *Report the dependent variable and connectivity measure, specifying weighted graph or binarized graph, subject- or group-level, and the global and/or node summaries used (e.g. clustering coefficient, efficiency, etc.).*

Multivariate modeling and predictive analysis | *Specify independent variables, features extraction and dimension reduction, model, training and evaluation metrics.*
